# Beneficial effect of fluoxetine treatment aganist psychological stress is mediated by increasing BDNF expression in selected brain areas

**DOI:** 10.18632/oncotarget.17891

**Published:** 2017-05-15

**Authors:** Gongying Li, Ping Jing, Zhidong Liu, Zhiruo Li, Hongxia Ma, Wenzhen Tu, Wei Zhang, Chuanjun Zhuo

**Affiliations:** ^1^ Insitute of Mental health, Jining Medical University, Jining, 272067, China; ^2^ Department of Psychiatry, Wenzhou Seventh People’s Hospital, Wenzhou, Zhejiang Province, 325000, China; ^3^ Tianjin Fourth Central Hospital of Tianjin Medical University, Tianjin, 300000, China; ^4^ Department of Neurology, The Affiliated Hospital of Jining Medical University, Jining, 272000, China; ^5^ Department of Psychiatry, The Second Affiliated Hospital of Jining Medical University, Jining, 272051, China; ^6^ Department of Psychiatric Neuroimaging Laboratory, Tianjin Anding Hospital, Tianjin Mental Health Center, Tianjin 300222, China; ^7^ Department of Psychiatry, Tianjin Anning Hospital, Tianjin 300300, China

**Keywords:** brain-derived neurotrophic factor (BDNF), psychological stress, fluoxetine, serotonin

## Abstract

SSRI antidepressant fluoxetine is widely used to treat psychological stress related disorders, however the underlying working mechanisms is not fully understood, as SSRIs can rapidly increase the extracellular serotonin levels but it normally takes weeks to reveal their therapeutic effect in the stress-related psychological disorders. Our previous study demonstrated that purely psychological stress without any physic stimuli induces a biphasic change in the expression of brain-derived neurotrophic factor (BDNF), which immediately decrease and then gradually increase after the stress; and that the latter BDNF increase in response to the psychological stress involves the activation of serotonin system. To investigate the role of BDNF in the fluoxetine treatment for stress-related psychological disorders, we examined the mRNA and protein levels of BDNF in the brain of Sprague-Dawley (SD) rats, which were pretreated with fluoxetine at 10 mg/kg or vehicle solution for 14 days, over 24 hour after an acute psychological stress exposure. In situ hybridization and immunohistochemistry were performed to detect the expression of BDNF at different time points in various brain regions after the psychological stress. We found that fluoxetine treatment completely blocked the BDNF decrease induced by the psychological stress, and also enhanced the gradual increase in the expression of BDNF in most of the brain regions except VTA after the psychological stress. The results suggest that the enhancement in BDNF levels induced by chronic fluoxetine treatment mediates the therapeutic effect against psychological stress.

## INTRODUCTION

Acute and chronic psychological stress can provoke life-threatening diseases, such as depression and post-traumatic stress disorder [1]. Understanding the pathological response and underlying biological mechanisms of psychological stress would promote the discovery of efficient treatments for psychological stress and stress-related disorders. Besides resulting negative affective states that could induce toxic biological processes and further influence disease risk [2, 3], psychological stress could also engage adaptive protective pathways such as to boost and relocate energy through hypothamalmic-pituitary-adrenal (HPA) axis to the “battle station” and to active immune defense system to limit or avoid the adverse impact of stressor [4–6]. Been long studied, neurotrophic factors were shown to be important mediators of stress responses, Brain Derived Neurotrophic Factor (BDNF) in particular [7–9]. They can protect the organism from stress-induced aversive processes leading to diseases by preventing neuronal damage and facilitating neuronal growth as well as the neuroplasticity for memory formation. The changes of BDNF depends upon different types of stress [10–12], and varies in different brain areas [13–15]. Despite the variance, a down-regulation of BDNF was often observed after stress exposures in both preclinical and clinical studies [13, 16–19]. However, the stress models used in most of these studies with adult animals involved physical meddling of the animals, such as body restrain or electric foot shock. These models therefore can hardly represent purely psychological stress, which are more relevant in human societies. By applying communication box (CB) paradigm, a mode of purely psychological stress without physical stimulation, we recently demonstrated that, similar with other stressors, BDNF is indeed involved in psychological stress response with a biphasic change: the levels of BDNF in most brain areas decreased immediately after the shock, but were gradually recovered at about 2 hours after the shock and then increased within at least 24 hours after the psychological stress compared with a non-stressed control group [20]. It is widely accepted that the regulation of BDNF expression in response to stress is mediated by glucocorticoid [13, 21], but not entirely [7]. Using the same CB paradigm, we demonstrated the involvement of serotoninergic neurotransmission in the BDNF regulation in purely psychological stress response. The blockage of serotonin receptors 5-HT1A or 5-HT1B suppress the later increase of BDNF in most of brain areas after stress exposure; and the pre-treatments with 5-HT1A or 5-HT2A receptor agonists completely blocked the immediate decrease of BDNF after stress exposure and significantly enhanced the BDNF increase in the later phase in response to stress [22]. Previous studies suggested that serotonin release and 5-HT1A receptor activation are also involved in the perception and neuroadaptation in response to stressful stimuli [23–25]. In point of fact, serotonin and BDNF interact at multiple levels in the brain with strict temporal, 5-HT receptor subtype and spatial specificities [26].

Fluoxetine is a typical selective serotonin reuptake inhibitor (SSRI), widely used to treat the stress-related psychiatric disorders, such as major depression, obsessive-compulsive disorder (OCD), panic disorder and other neuropsychiatric disorders. Fluoxetine is also suggested to be an effective medication to improve anxiety, to prevent relapse in post-traumatic stress disorder (PTSD), another stress-related psychiatric disorder in adult [27–29]. Fluoxetine treatment in patient with PTSD during symptom provocation was reported to normalize the alteration of neural activity in prefrontal and paralimbic cortices, which are involved in memory, emotion, attention and motor-control [30]. Preclinical studies also showed that fluoxetine could reverse the aversive impacts of psychological stress, such as reducing fear and anxiety behaviors resulted from an early life stress -- maternal separation [31]; ameliorating risk assessment behaviors induced by chronic mild stress [32, 33]; preventing the increased anxiety level, enhanced HPA axis inhibition, impaired inhibitory avoidance conditioning and extinction induced by single prolonged stress (SPS), which is a validated animal model for PTSD [34]. However, the working mechanism of this antidepressant is not fully understand. Fluoxetine can rapidly increase serotonin levels in extracellular space, but most of its antidepressant effects take place after weeks of treatment. This suggests that gradual adaptation downstream pathways are likely involved. As supporting evidence, one recent study discovered that in a social defeat model fluoxetine induces epigenetic modifications at the promoter of protein kinase calmodulin-dependent protein kinase II (CaMKII) gene in nucleus accumbens (NAc), maybe through the activation of serotonin receptors in NAc [35]. The results from other recent studies using a SPS model and a forced swimming stress model point towards another possible mechanism involved in the fluoxetine treatment for stress induced symptoms: anti-apoptotic and neuroprotective effect through increasing BDNF expression in the brain [34, 36]. The reviews from Duman’s group also suggested that the beneficial effects of fluoxetine in alleviating stress-related disorders was through increasing BDNF expression [9, 12, 37]. Considering our research result showing that 5-HT receptor activation enhances the BNDF expression in the brain, the SSRI antidepressant fluoxetine may enhance the BNDF expression by increasing the serotonin level in the synaptic cleft and activating serotonin receptors. Moreover, the augmented levels of BDNF could retrogradely induce regenerative serotoninergic axonal sprouting and dendrites expanding in hippocampus and in other brain areas such as amygdala to alleviate stress-related psychiatric symptoms [38–40]. Thus, the reciprocal action between serotonin and BDNF may well synergize the pharmacological effect of fluoxetine.

To investigate this hypothesis, in this study, we examined the effect of a chronic pre-treatment of fluoxetine on the BDNF expression in response to a purely psychological stress by using CB paradigm. The result could lead us not only to understand better the working mechanisms of fluoxetine, but also to identify novel therapeutic measures for psychological stress and related disorders through the BDNF pathway.

## RESULTS

### BDNF mRNA expression after psychological stress and fluoxetine treatment

**Figure 1:**
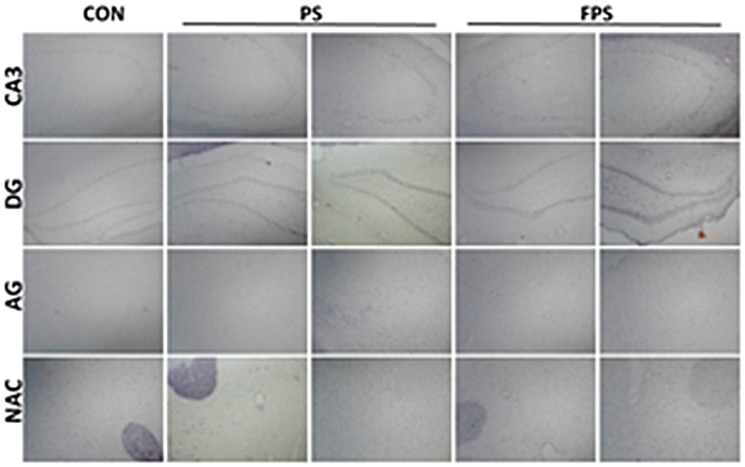
Representative images of ISH staining of BDNF in various brain regions at 0 or 24 hours after psychological stress exposure with or without fluoxetine treatment

To investigate the regulation of BDNF mRNA in three animal groups respectively receiving: 1) non psychological stress but pre-treated with vehicle control (CON) for 14 days; 2) psychological stress (PS); and 3) psychological stress and pre-treated with fluoxetine at 10 mg/kg for 14 days (FPS), we analyzed the mRNA expression of BDNF by using *in situ* hybridization (ISH) with *bdnf* oligo-probe in various brain regions, which included hippocampus brain regions of CA1 and CA3, dentate gyrus (DG), prefrontal cortex (PFC), shell of nucleus accumbens (NAc), central amygdaloid nuclei (AG), midbrain periaqueductal gray (PAG), dorsomedial hypothalamic nucleus (DM) and ventral tegmental area (VTA) over 24 hours after the stress exposure (Figure 1).

**Figure 2:**
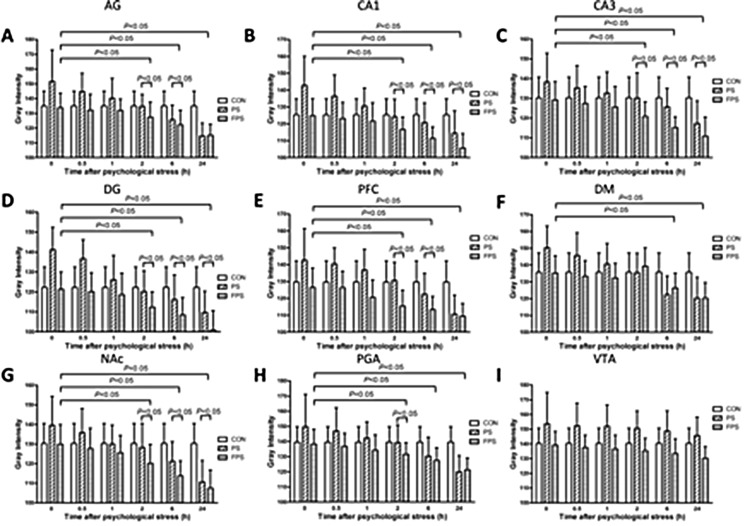
255-grey intensity quantification of BDNF mRNA expression at different time points after psychological stress exposure in the selected brain regions in the CON, the PF and the FPS groups BDNF mRNA expression, negatively correlated with the gray intensity values, were quantified from ISH staining in different brain regions including AG (**A**), hippocampus CA1 (**B**), CA3 (**C**), DG (**D**), PFC (**E**), DM (**F**), NAc (**G**), PAG (**H**), and VTA (**I**) at 0, 0.5, 1, 2, 6 and 24 hours after psychological stress exposure. Data are presented as mean ± SE, n=5 for each group at each time point. Data were analyzed by two-way ANOVA followed by a post hoc test using the least significance difference method (LSD). *P* < 0.05: significant difference between the marked two groups or subgroups.

The gray intensity analysis confirmed our previous result [20] that the gray intensity of the BDNF ISH staining of the PS group significantly differed from that of the CON group in most of the examined brain areas (in CA1: F_[2,12]_ = 8.907, *p =* 0.004; in CA3: F_[2,12]_ = 6.291, *p =* 0.014; in DG: F_[2,12]_ = 11.153, *p =* 0.002; in PFC: F_[2,12]_ = 8.884, *p =* 0.004; in NAc: F_[2,12]_ = 5.405, *p =* 0.021; in AG: F_[2,12]_ = 8.971, *p =* 0.004; in PAG: F_[2,12]_ = 5.898, *p =* 0.016; in DM: F_[2,12]_ = 7.353, *p =* 0.008) except in VTA (F_[2,12]_ = 1.515, *p =* 0.259), however, the gray intensity of FPS and CON was not differed significantly in all brain regions,as shown in Figure 2. Moreover, post-hoc analysis demonstrated that the change in BDNF mRNA levels in PS group was a biphasic modulation: immediately after the psychological stress, the gray intensity of BNDF ISH staining in the PS group was significantly increased and started to decline; after 2 hours, the gray intensity of the PS group became lower than that of the CON group. This result means that psychological stress in CB paradigm induced a biphasic change of BDNF mRNA: an immediate decrease followed by a gradual increase last for at least 24 hours, which is in accordance with our previous observation [20].

While for the animals treated with fluoxetine (FPS), the gray intensity was significant lower than that of the PS group in all the brain areas The pairwise multiple comparison procedures using Bonferroni *t*-test demonstrated that the gray intensity of PS was significant higher than that of the FPS in the examined brain areas (in CA1: t =3.307, *p =* 0.009; in CA3: *t =* 3.254, *p =* 0.021; in DG: *t =* 4.180, *p =* 0.004; in PFC: *t =* 3.976, *p =* 0.006; in NAc: *t =* 2.897, *p =* 0.040; in AG:*t =* 3.785, *p =* 0.008; in PAG: *t =* 3.129, *p =* 0.026) and that of the CON in the examined brain areas (in CA1: t =3.606, *p =* 0.011; in CA3: *t =* 2.864, *p =* 0.043; in DG: *t =* 3.994, *p =* 0.005; in PFC: *t =* 3.201, *p =* 0.023; in NAc: *t =* 2.795, *p =* 0.049; in AG:*t =* 3.540, *p =* 0.012; in PAG: *t =* 2.791, *p =* 0.049; in DM: *t =* 3.263, *p* = 0.020), and also lower than (/but not different from) that of the CON group over 24 hours after the stress. Post-hoc analysis shows that there was no significant change in gray intensity of BDNF ISH staining in PS group until 2 hours after the stress exposure in most of the examined brain areas except in VTA. This result suggests that chronic fluoxetine treatment could block the immediate decrease in BDNF mRNA induced by the psychological stress in our experimental model and significantly up-regulate the mRNA expression of BDNF in the brain despite the psychological stress, and this elevation in BDNF mRNA lasted at least till 24 hours after the psychological stress. However, there was no significant difference between BDNF mRNA levels in FPS and PS groups in PFC, DM and PAG brain regions at the 24 hour time point (Figures 1 and 2).

### BDNF protein expression after psychological stress and fluoxetine treatment

**Figure 3:**
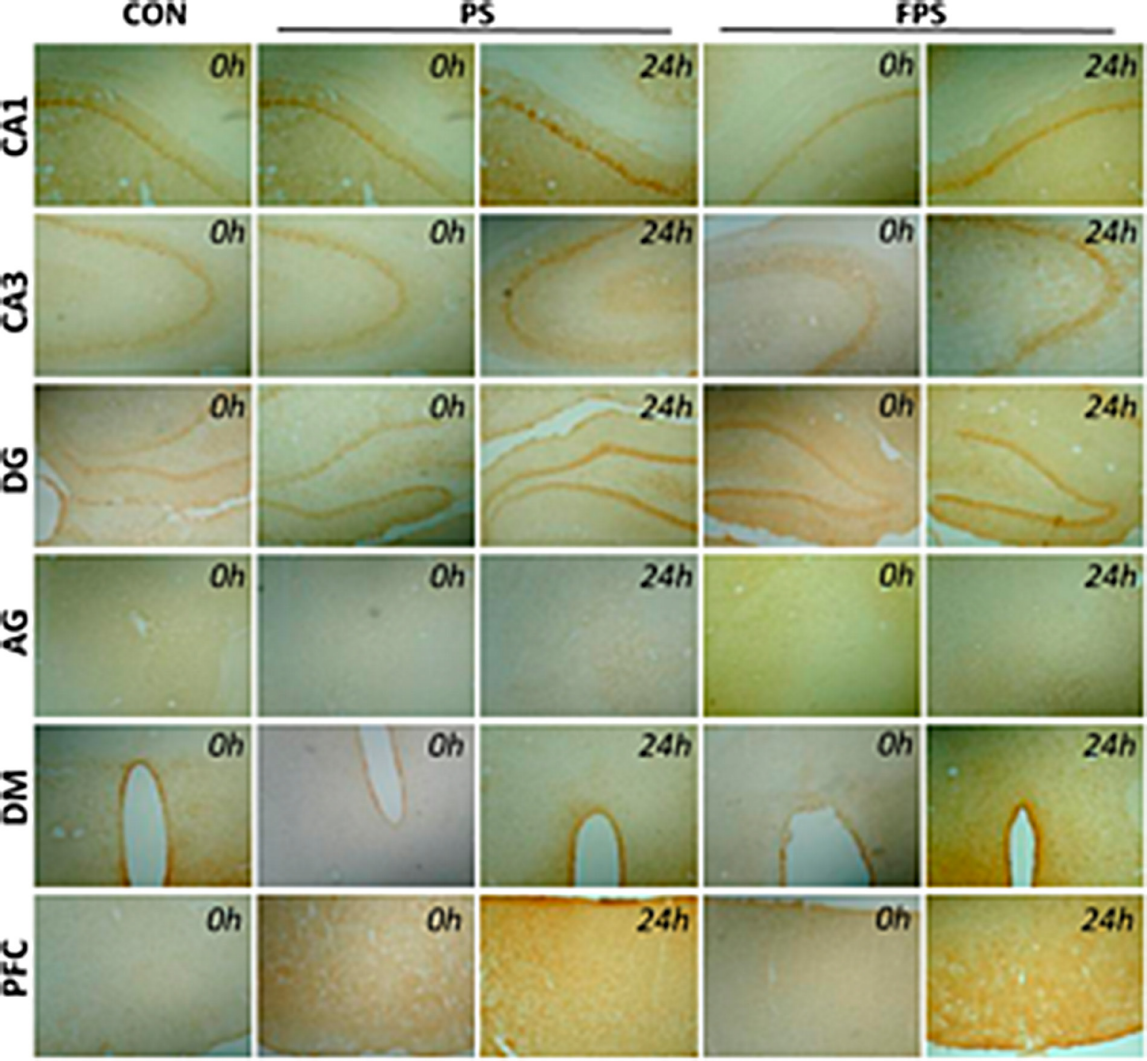
Representative images of BDNF immunohistochemical staining in various brain regions at 0 or 24 hours after psychological stress exposure with or without fluoxetine treatment

To obtain a functional BDNF protein, the bioprocess from BDNF mRNA includes the translation to a pro-BDNF protein and post-translational modifications to the mature form of BDNF, which is a complicated process [41]. The mRNA levels are not always correlated to the protein levels. We therefore investigate the regulation of BDNF protein levels by the purely psychological stress with and without the treatment of the fluoxetine in this study. The protein expression of BDNF is analyzed by using immunohistochemistry (IHC) staining in the same brain regions of the animas of the CON, the PS and the FPS three groups as in the mRNA analysis (Figure 3).

**Figure 4:**
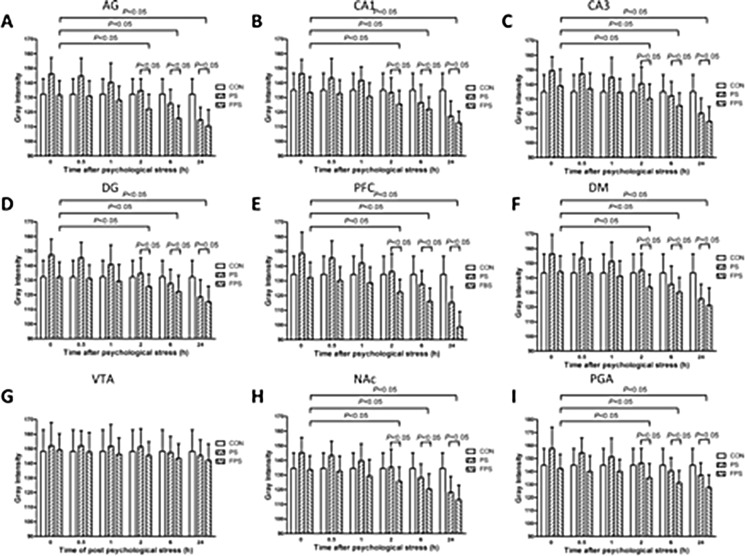
255-grey intensity quantification of BDNF protein expression at at different time points after psychological stress exposure in various brain regions in the CON, the PS and the FPS groups BDNF protein expression, negatively correlated with the gray intensity values, were quantified from IHC staining in different brain regions including AG (**A**), hippocampus CA1 (**B**), CA3 (**C**), DG (**D**), PFC (**E**), DM (**F**), VTA (**G**), NAc (**H**), and PAG (**I**) at 0, 0.5, 1, 2, 6 and 24 hours after psychological stress exposure. Data are presented as mean ± SE, *n* = 5 for each group at each time point. *P* < 0.05: significant difference between the marked two groups or subgroups.

The gray intensity analysis of IHC staining showed the same trend as in the ISH result:

a) Psychological stress significantly changed the BDNF protein expression, compared with the CON group, in most of the examined brain areas (in CA1: F[2,12] = 6.300, *p =* 0.013; in CA3: F[2,12] = 6.065, *p =* 0.015; in DG: F[2,12] = 10.997, *p =* 0.002; in PFC: F[2,12] = 10.382, *p =* 0.002; in NAc: F[2,12] = 5.787, *p =* 0.017; in AG: F[2,12] = 5.82, *p =* 0.017; in PAG: F[2,12] = 6.53, *p =* 0.012; in DM: F[2,12] = 5.232, *p =* 0.023xx) except in VTA (F[2,12] = 0.107, *p =* 0.899), as shown in Figure 4. It is also a biphasic modulation: the stress induced immediate significant decreases in BDNF protein levels, which was shown as increases in the gray intensity, in the PS group compared with that in the CON group; and the BDNF protein levels in the PS group started to increase, shown as decrease in the gray intensity, over 24 hours after the stress, it became higher (shown as lower in gray intensity) than the BDNF protein levels in the CON group after 2 hours.

b)The pre-treatment of fluoxetine for 14 days showed a significant up-regulation of BDNF protein levels (down-regulation in the gray intensity graphs) in most of the brain regions except VTA over 24 hours after psychological stress exposure (FPS), compared with both PS only group The pairwise multiple comparison procedures using Bonferroni *t*-test demonstrated that the protein expression of FPS was significant higher than that of the PS in the examined brain areas (in CA1: *t =* 3.254, *p =* 0.021; in CA3: *t =* 2.972, *p =* 0.035; in DG: *t =* 4.061, *p =* 0.005; in PFC: *t =* 4.186, *p =* 0.004; in NAc: *t =* 3.044, *p =* 0.031; in AG:*t =* 3.006, *p =* 0.033; in PAG: *t =* 3.378, *p =* 0.016) and that of the CON in the examined brain areas (in CA1: *t =* 2.855, *p =* 0.043; in CA3: *t =* 3.058, *p =* 0.030; in DG: *t =* 4.061, *p =* 0.005; in PFC: *t =* 3.652, *p =* 0.010; in NAc: *t =* 2.838, *p =* 0.045; in AG:*t =* 2.901, *p =* 0.040; in PAG: *t =* 2.801, *p =* 0.048; in DM: *t =* 2.887, p=0.041), however, the protein expression of FPS and CON was not differed significantly in all brain regions. The post-hoc comparison indicated that the BDNF protein levels in most of the brain regions except VTA in the FPS group also did not start to significantly increase (decrease in gray intensity) until 2 hours after the stress, the increases lasted for at least till 24 hours after the stress. The result suggested that fluoxetine treatment could also completely block the psychological stress-induced immediate BDNF protein decrease (increase in gray intensity) (Figures 3 and 4) and further enhance the BDNF protein increase in response to the psychological stress in most of the brain areas, but not in VTA.

## DISCUSSION

The current study investigated the effect of fluoxetine treatment on the expression of BDNF protein and mRNA in various brain regions in the CB stress model. Confirming with our previous results [20], the result of this study showed that the acute purely psychological stress resulted an immediate attenuation of BDNF mRNA and protein levels in most of the brain regions except VTA in the PS group in comparison with the CON group; and the BDNF expression, both in mRNA and protein, in the PS group gradually increased over 24 hours after the stress. Our result in this study also showed that the expression levels of BDNF protein and mRNA in the fluoxetine-pretreated group (FPS) were significantly higher than those in the PS group, suggesting that the fluoxetine treatment increased the expression of the BDNF protein and mRNA in various brain areas after the acute psychological stress. In addition, the prior chronic treatment of fluoxetine completely abolished the immediate attenuation of BDNF induced by the stress, and further enhanced both the mRNA and the protein expression of BDNF in most of the brain regions but not in VTA after the stress exposure.

Our result of the effect of fluoxetine on the BDNF expression is in accordance with the studies applying other stress models. Such as Haynes *et al.* found that a daily intraperitoneal injection of 8 mg/kg fluoxetine in rats continuously for 10 days significantly increased BDNF expression in the hippocampal and striatal regions with dexamethasone-induced neuronal damage [42]. To our knowledge, this is the first study directly showing the effect of fluoxetine on the BDNF expression in an acute purely psychological stress model, which is more relevant to the psychological stress encountered in human society. Combined with previous studies, our findings demonstrated that the beneficial effect of fluoxetine on the stress related disorders, at least partially, is due to the up-regulation of the BDNF expression in the brain. Moreover, our results suggested that increasing BDNF expression could be an alternative therapeutic strategy for the treatment of stress-related psychological disorders.

Psychological stress exposure triggers hypothalamic-pituitary-adenocortical (HPA) axis activation and associated neurotransmission modulation, primarily in limbic pathway including hippocampus, amygdala and connections to the prefrontal cortex (PFC), which are enriched with glucocorticoid receptors [43, 44]. Hippocampus and amygdala convey respectively context and emotional information to the nucleus accumbens (NAc) in response to psychological stress [45]. In additional, midbrain periaqueductal gray (PAG) receiving output from amygdala was also involved in the behavioral responses to uncontrollable stress [46]. While it is well known that dopamine (DA) neurons in ventral tegmental area (VTA) is prone to be activated by rewording stimuli, aversive events or stress also excites certain DA neurons in the VTA [47]. Moreover, the therapeutic effects of SSRI antidepressant also involve all the aforementioned brain regions: hippocampus brain regions of CA1 and CA3, dentate gyrus (DG), PFC, NAc, central amygdaloid nuclei (AG), PAG, dorsomedial hypothalamic nucleus (DM) and VTA that receive serotoninergic input from raphe nuclei [48,49]. Except in VTA, the fluoxetine pre-treatment in this study showed comparable effects on the BDNF mRNA expression and almost the same effect on the BDNF protein expression in the brain regions mentioned above after psychological stress: the immediate decrease in BDNF expression induced by the stress was completely blocked and the gradual BDNF increase after the stress was significantly boosted by the fluoxetine. As discussed above, the VTA is the least involved in the response to psychological stress among all the examined brain regions, it is not surprising that little change of BDNF was observed in this area. It is worth to mention that between the mRNA and the protein of BDNF, the protein is the actual growth factor and functional neuroprotective molecule, it can further react with the BDNF receptor tyrosine receptor kinase B (TrkB) to trigger its downstream process to promote neuronal survival, plasticity, axonal growth and anti-apoptosis [50, 51].

Some previous studies provided insights on the molecular mechanisms that could be involved in the enhancement of BDNF expression induced by chronic fluoxetine treatment. Chronic application of fluoxetine was shown to selectively react with 5-HT1A receptor [52, 53], enhancing the receptor-G protein capability of 5-HT1A receptor [54, 55]. Increased extracellular serotonin resulted by fluoxetine inhibiting serotonin uptake acts on the 5-HT1A receptor, which could in turn activate the cAMP-protein kinase A (PKA) pathway through the G protein activation, and subsequently activate certain transcription factors such as CREB, which is known to promote the expression of neurotrophic factor BDNF [56–58]. Together with our recent finding [22], it is suggested that fluoxetine treatment could regulate the expression of BDNF protein through the 5-HT1A and 5-HT2A receptor pathways.

There are a few limitations in this study: 1) The animals in the CON group and the FPS group received once daily intraperitoneal (i.p.) injection of vehicle solution or fluoxetine for 14 consecutive days, while the animals in the PS group did not receive any injection before the psychological stress. The i.p. injection and animal handling might induce physical stress in the experimental animals. Although after 14 days, the animals probably became habituated to the i.p. injection, the physical stimulation thus may have little effect of stress. A subcutaneous implantable infusion device for chronic drug release may be applied to avoid the possible physical stress associated with i.p. injection, we will look into this drug administration method in our future studies. 2) We did not include a fluoxetine control non-stress group in the present study. In one previous study, de Foubert *et al.* showed that the expression of BDNF protein in the animals who received chronic oral administration of the same dose of fluoxetine used in our present study (10 mg/kg) for also 14 days, was significantly increased compared with their vehicle control group [59]. We will nevertheless include a fluoxetine-treated control non-stress group in our future studies. 3) The gray intensity analysis used in this study is a bit outdated, in the future, we will perform RT-PCR and western blot assay to determine the expression levels of BDNF mRNA and protein, respectively.

In summary, our findings suggest that chronic fluoxetine treatment can significantly up-regulate the expression of BDNF protein and mRNA in the brain after an acute psychological stress exposure, the reciprocal action between 5-HT and BDNF could further synergize the therapeutic effect of fluoxetine. This study also provides a supporting evidence for a novel treatment strategy of increaseing BDNF expression for the stress-related disorders.

## MATERIALS AND METHODS

### Ethics statement

This study was carried out in strict accordance with the recommendations in the Guide for the Care and Use of Laboratory Animals of Central South University. The protocol was approved by the Committee on the Ethics of Animal Experiments of The Second Xiangya Hospital, Central South University (No.2005-R99).

### Animal treatment

Sixty-five adult male Sprague–Dawley (SD) rats (inbred strain, Animal Center, The Second Xiangya Hospital, Central South University, China) weighing 180–220 g were used for the following experiments. Animals were housed four animals per cage in standard polycarbonate cages with free access to food and water, with a 12/12 hr light/dark cycle and a temperature-regulated environment (23 ± 1°C).

The animals in this study were divided into three major groups: fluoxetine psychological stress (FPS) group, blank psychological stress (PS) group, and vehicle solution control non-stress (CON) group. The FPS group and PS group were each further divided into six sub-groups according to the following examination time points after the stress exposure: 0 (immediate), 0.5, 1, 2, 6, and 24 hours, each sub-group was composed of five rats; CON group included 5 rats. Groups of five male SD rats from a total of 65 male rats were randomly assigned into each group or sub-group. *CON group treatment*: the rats in this group received intraperitoneal (i.p.) injections of 0.9% physiological saline once daily at 9:00 am for 14 consecutive days. This group did not receive any psychological stress. *FPS group treatment*: Pure grade fluoxetine (Changzhou Watson Pharmaceutical Co. Ltd.) was dissolved in 0.9 % saline, and once daily i.p. injection of 10 mg/kg was given to each rat for 14 consecutive days as previously described [53]. Psychological stress was given on the 15^th^ day once daily for two days using the communication box (CB) paradigm stress method. *PS group treatment*: The rats in this group did not receive any injection of drugs or reagents and only two psychological stress procedures were given once daily for two days. All the animals were terminated at the designated examination time point after the last psychological stress event was completed.

### Communication box (CB) paradigm

A CB apparatus was modified from a previously reported protocol [60]. The CB is characterized by the complete removal of physical stimuli from the responder rats. Psychological stress in the responder rats is induced solely by communication between the responder rats and the sender rats. The apparatus used for this study consisted of a box with wooden walls that measured 60 cm in width, 60 cm in length and 44 cm in height. The floor of the apparatus consisted of a grid of stainless steel rods, 5 mm in diameter and spaced 1 cm apart, center to center. The box interior was divided into nine compartments with transparent Plexiglas walls. Each compartment measured 20 cm in length and width and 44 cm in height. Each Plexiglas wall had a single hole (6 cm from the floor, 2 cm in diameter). An electric shock (1.5 – 2.2 mA) was delivered to the floor of the center“sender rat” compartment with a shock generator. A thick insulated plate was placed on the floor of the “responder rat” compartments to prevent foot shock. The animals placed in the sender rat compartment responded to the foot shocks with squeals, jumps, piloerections and defecation. The animals in the responder rat compartments were influenced by the visual, auditory and olfactory response of the senders, but they did not receive any direct physical stimuli.

### Sample preparation

At each time point after stress, the animals were anaesthetized with pentobarbital sodium via i.p. injection, followed by intracardiac perfusion of 150 ml sterile saline and then 250 ml of 4% paraformaldehyde. Brain tissues were immediately collected after perfusion, then post-fixed with the same fixative and properly stored till brain slice preparation. Continuous coronal sections were obtained using a cryostat frozen section machine (American HistoSTAT MicroTOME) at −20°C. The sections were 30 μm thick. With the reference to rat anatomy, specimens containing the hippocampus brain regions of CA1, CA3, dentate gyrus (DG), prefrontal cortex (PFC), central amygdaloid nuclei (AG), shell of nucleus accumbens (NAc), midbrain periaqueductal gray (PAG), ventral tegmental area (VTA), and dorsomedial hypothalamic nucleus (DM) were retained.

### *In situ* hybridization (ISH)

The ISH experiment was performed according to the manufacturer’s protocol using BDNF mRNA expression detection kits purchased from Wuhan Boster Biological Technology, Ltd. (Wuhan, China). The sequence of the BDNF oligo-probe was: 5′-GCT GAG CGT GTG TGA CAG TAT TAG TGA GTG-3′. In brief, brain sections were mounted on poly-L-lysine coated slides, endogenous peroxidase was inactivated, the sections were pre-hybridized followed by an incubation with digoxin-labeled BDNF oligo-probes at 37°C for 14 hrs. After washing, the sections were incubated with biotinylated mouse anti-digoxin antibodies at 37°C for 60 min, and then with a streptavidin-biotin-peroxidase complex for 20 min, followed by an additional incubation with biotinylated peroxidase for 20 min. Finally, the sections were developed, mounted and cover-slipped. All the experimental procedures were performed under strict RNAse-free conditions with autoclaved instruments and solvents. Controls were arranged on adjacent sections to ensure the specificity of the probe. Control sections were firstly treated with RNAase, followed by the ISH procedures described above in the presence or absence of the oligonucleotides probe.

### Immunohistochemistry (IHC) staining

The IHC experiment in this study was modified from the standard protocol. The brain sections were incubated with the primary antibody of BDNF (1:100; Santa Cruz Biotechnology, CA, USA) at 4**°**C overnight. After rinsed, the sections were incubated with biotinylated secondary antibody (1:100; Santa Cruz Biotechnology) for 2 hrs. After the sections were washed, ABC compound (1:100) was added to the sections and incubated for 2 hours at room temperature. DAB was finally applied to develop color. After the extra DAB was rinsed off, the sections was dehydrated and mounted. The negative control sample was prepared by replacing the primary antibody with normal goat serum, and with the remaining steps unchanged.

### Gray intensity analysis

BDNF signal quantification was achieved with the aid of a computerized video-imaging system (HPIAS-1000, Wuhan Champion Image Engineering Co. Ltd., Wuhan, China) by determining the 255-gray intensity of BDNF mRNA or protein expression on each section with the targeted brain regions. Sections of each group were analyzed under the same ISH or IHC conditions. For each animals, four brain sections containing the targeted brain region were randomly selected, and four fields from each section in the targeted brain region were randomly selected for analysis. Gray intensity was measured at the same anteroposterior level in each targeted brain region. The gray intensity value reflected the relative expression level of protein or mRNA, which was negatively correlated with the gray intensity values.

### Statistical analysis

All test results unless otherwise stated are expressed as the means ± standard error (SE). A two-way ANOVA was used for analyze the difference in the mRNA or protein expression of BDNF at different examination time points and in different groups. If there was a significant difference, a post-hoc test with Fisher’s least-significant difference (LSD) method would be applied. Differences were consider significant at *P < 0.05.* All statistical analyses were performed using SPSS 11.5 software package.
